# Gene Expression Profiles of Human Cerebral Organoids Identify PPAR Pathway and *PKM2* as Key Markers for Oxygen-Glucose Deprivation and Reoxygenation

**DOI:** 10.3389/fncel.2021.605030

**Published:** 2021-06-08

**Authors:** Naoki Iwasa, Takeshi K. Matsui, Naohiko Iguchi, Kaoru Kinugawa, Naritaka Morikawa, Yoshihiko M. Sakaguchi, Tomo Shiota, Shinko Kobashigawa, Mari Nakanishi, Masaya Matsubayashi, Riko Nagata, Sotaro Kikuchi, Tatsuhide Tanaka, Nobuyuki Eura, Takao Kiriyama, Tesseki Izumi, Kozue Saito, Hiroshi Kataoka, Yuichi Saito, Wataru Kimura, Akio Wanaka, Yuhei Nishimura, Eiichiro Mori, Kazuma Sugie

**Affiliations:** ^1^Department of Neurology, Nara Medical University, Kashihara, Japan; ^2^Department of Future Basic Medicine, Nara Medical University, Kashihara, Japan; ^3^Department of Anatomy and Neuroscience, Nara Medical University, Kashihara, Japan; ^4^Laboratory for Heart Regeneration, RIKEN Center for Biosystems Dynamics Research, Kobe, Japan; ^5^Department of Integrative Pharmacology, Mie University Graduate School of Medicine, Tsu, Japan; ^6^V-iCliniX Laboratory, Nara Medical University, Kashihara, Japan

**Keywords:** cerebral organoid, human induced pluripotent stem cells, oxygen-glucose deprivation/reoxygenation, ischemic stroke, pyruvate kinase M2, peroxisome proliferator-activated receptor, ferritin light chain

## Abstract

Ischemic stroke is one of the most common neurological diseases. However, the impact of ischemic stroke on human cerebral tissue remains largely unknown due to a lack of ischemic human brain samples. In this study, we applied cerebral organoids derived from human induced pluripotent stem cells to evaluate the effect of oxygen-glucose deprivation/reoxygenation (OGD/R). Pathway analysis showed the relationships between vitamin digestion and absorption, fat digestion and absorption, peroxisome proliferator-activated receptor (PPAR) signaling pathway, and complement and coagulation cascades. Combinational verification with transcriptome and gene expression analysis of different cell types revealed fatty acids-related PPAR signaling pathway and pyruvate kinase isoform M2 (*PKM2*) as key markers of neuronal cells in response to OGD/R. These findings suggest that, although there remain some limitations to be improved, our ischemic stroke model using human cerebral organoids would be a potentially useful tool when combined with other conventional two-dimensional (2D) mono-culture systems.

## Introduction

Stroke, a cerebrovascular disease, is a common neurological disorder, and ischemic strokes are among the major causes of permanent morbidity and disability (Spescha et al., 2013; Knowland et al., 2014; Benjamin et al., 2019). Several therapeutic agents are available for acute ischemic stroke, including thrombolytics, anticoagulants, and antiplatelets. However, their efficacy is restricted and their use is limited (Catanese et al., 2017). Mechanical thrombolytics and recombinant tissue plasminogen activator (rt-PA) have therapeutic time windows for hyperacute ischemic stroke, because the target of these therapies is to rescue the penumbra around the ischemic core (Fisher and Bastan, 2012; Dorado et al., 2014). Thus, elucidating the mechanism of ischemic stroke in human cerebral tissue is expected to be important for the development of appropriate therapies.

Thus far, two-dimensional (2D) neuron cultures have been utilized in ischemic models. However, there are limitations to these models as actual brain tissues are composed of multiple cell types and responses to ischemia take place from cell to cell. Recent progress in the development of organs in-a-dish (organoids) provides potential for the modeling of various diseases (Clevers, 2016). Organoids resemble the architecture of organs that are composed of multiple cell types, and three-dimensionally (3D) cultured cerebral organoids are expected to represent physiological environment (Pasca et al., 2015). In this study, we analyzed the gene expression profiles of human cerebral organoids after oxygen-glucose deprivation/reoxygenation (OGD/R).

## Materials and Methods

### Human Cerebral Organoids Generation and Culture

Human induced pluripotent stem cells (iPSCs) which were Cellartis Human iPS Cell Line 12 (ChiPSC12, Y00285, Takara Bio, Kusatsu, Shiga, Japan) were cultivated in mTeSRTM1 medium (05851, Stemcell Technologies, Vancouver, British Columbia, Canada) and maintained in feeder-free condition with mTeSR-TM1 media. Feeder-free human iPSC line (XY) from Takara was obtained on six well plates (3506, Corning, New York, USA) coated with growth factors reduced Matrigel (356230; BD Biosciences, San Jose, CA, USA). At the time of passage, we added Rho-associated protein kinase (ROCK) inhibitor (final concentration 10 μM; Selleck Chemicals, Houston, Texas, USA), and maintained these cells with daily medium change without ROCK inhibitor until they reached about 70% confluency. Then, they were detached by versene solution (15040-066; Thermo Fisher Scientific, Waltham, MA, USA) and seeded by a 1:20 dilution ratio.

Cerebral organoids were differentiated according to previously published protocol (Lancaster et al., 2013). Human iPSCs were detached and subjected to embryoid body (EB) induction using the protocol. After 4 days, half of the media was replaced by human EB medium without ROCK inhibitor and basic fibroblast growth factor (bFGF). Two days later, the EBs were transferred into a neural induction media which consisted of DMEM-F12 with 1× N2 supplement (17502048; Thermo Fisher Scientific, Waltham, MA, USA), 1× Glutamax, 1× non-essential amino acids, and heparin (1 μg/ml) and then embedded in Matrigel after 5 days. Subsequently, the organoids were induced in organoid medium by using an orbital shaker. The total process took 6 weeks, from EB formation.

### Immunohistochemical Analysis of Human Cerebral Organoids

Human cerebral organoids were fixed in 4% paraformaldehyde in Phosphate-Buffered Saline (PBS) overnight at 4°C, dehydrated with 30% sucrose in PBS and embedded in O.C.T. Compound (Thermo Fisher Scientific, Waltham, MA, USA). Cryostat sections (14 μm) were cut and mounted on slides (Thermo Fisher Scientific, Waltham, MA, USA). Mounted sections were incubated at room temperature for 1 h with blocking solution [3% normal goat serum + 0.3% Triton X-100 in tris-buffered saline (TBS)] and subsequently incubated with primary antibodies diluted in blocking solution overnight at 4°C. Antibodies specific for TUJ1 (1:400, T8660, Sigma-Aldrich, St. Louis, Missouri, USA) were used for immunostaining. After three washes with TBS, corresponding fluorescent dye Alexa Fluor 488-conjugated anti-mouse IgG (715-545-151, Jackson Immunoresearch, West Grove, PA, USA) secondary antibodies diluted in the blocking solution were added and samples were incubated at room temperature for 2 h and followed by 4′,6-diamidino-2-phenylindole (DAPI) (Vectashield Mounting Medium with DAPI, H-200; Vector Laboratories, Burlingame, CA, USA) staining. Finally, stained slides were rinsed with TBS three times, mounted, and analyzed using a FV3000 Confocal Microscope (Olympus, Shinjuku, Tokyo, Japan).

### Oxygen-Glucose Deprivation/Reoxygenation and RNA Extraction of Human Cerebral Organoids

The organoids were plated in Dulbecco's Modified Eagle Medium without glucose [DMEM (No Glucose), 09891-25; NACALAI TESQUE, Kyoto, Kyoto, Japan], and 1% penicillin-streptomycin (26253-84; NACALAI TESQUE, Kyoto, Kyoto, Japan). Subsequently, the organoids were cultured in a hypoxic incubator (94% N_2_, 5% CO_2_, 1% O_2_) at 37°C for 1 h. For reoxygenation, the medium was changed to Primary Neuron Basal Medium, then incubated (95% air, 5% CO_2_) at 37°C for 1 h. RNA from human cerebral organoids was extracted according to the manufacturer's protocol supplied with TRIzol reagent (15596018; Thermo Fisher Scientific, Waltham, MA, USA). The concentration and purity of the RNA samples were measured using Spectrophotometer (Beckman Coulter, Brea, California, USA).

### RNA Sequencing of Human Cerebral Organoids

Total RNA was isolated from the cells using the PureLink RNA Mini Kit (12183020; Thermo Fisher Scientific, Waltham, MA, USA) and according to the manufacturer's instructions. RNA concentration was analyzed by Qubit RNA HS Assay Kit (Thermo Fisher Scientific, Waltham, MA, USA) and the purity was assessed using the Qsep100 DNA Fragment Analyzer and RNA R1 Cartridge (BiOptic, New Taipei City, Taiwan). For RNA-sequencing (RNA-seq) analysis, each extracted RNA sample of non-treated organoid in equal concentration was adopted, and the same was adopted for OGD/R. Extracted RNA samples were either sent to a Bioengineering lab for RNA-sequencing analysis, and or subjected to RT-PCR. Then, total RNA was converted to cDNA and used for Illumina sequencing library preparation, according to KAPA Stranded mRNA-Seq Kit protocols (KAPA Biosystems, Wilmington, MA, United States). DNA fragments were ligated by FastGene Adapter Kit (NIPPON Genetics, Bunkyo, Tokyo, Japan). After the purified cDNA library products were appreciated using Qubit dsDNA HS Assay Kit (Thermo Fisher Scientific, Waltham, MA, USA), they were qualitatively evaluated using the Fragment Analyzer and dsDNA 915 Reagent Kit (Advanced Analytical Technologies) before it was finally sequenced (2 × 76 bp) on NextSeq 500 (Illumina, San Diego, CA, United States). Sickle (ver.1.33) is an error correction tool for quality trimming. It discards reads based upon the length threshold derived from quality score for FASTQ files. Using Sickle (ver.1.33), the threshold identified were quality values under 20 base and reads under 30 bp. Additionally, Hisat2 software was run with default parameters. The sequence alignment files generated by Sickle and Hisat2 were used to generate counts of mapped reads.

### Differentially Expressed Genes Extraction of Human Cerebral Organoids

By comparing organoids under OGD/R condition to non-treated organoids and using R package edgeR (Robinson et al., 2010) in R software (version 3.5.3), differentially expressed genes (DEGs) were extracted. The MA plot in R is a visual tool for showing the total gene expression levels of DEGs. The X-axis represents log CPM—log counts per million—and are measures of gene expression level. The Y-axis indicates log FC. Log FC is the log fold-change, which in this case, is the log difference between cerebral organoids after OGD/R.

### Gene Ontology and Pathway Analysis of Human Cerebral Organoids

Using the Database for Annotation, Visualization, and Integrated Discovery [DAVID, version 6.8 database (https://david.ncifcrf.gov/)], selected DEGs were analyzed. The DAVID shows the molecular function, biological process, and cellular component expressed in the gene profile. In this study, DAVID was applied to evaluate gene ontological (GO) annotation and Kyoto encyclopedia of genes and genomes (KEGG) pathways of DEGs. *P*-value < 0.05 was chosen as the threshold of KEGG pathway. Upregulation and downregulation of genes were analyzed separately.

### Protein–Protein Interaction Analysis of Human Cerebral Organoids

Based on the Search Tool for the Retrieval of Interacting Genes (STRING, https://string-db.org/; Szklarczyk et al., 2019) online database, DEGs were inputted, and the protein–protein interaction (PPI) network was constructed. The STRING database shows the interaction of each protein from prediction or experiments. The threshold to construct the PPI network was confidence score >0.4.

### Reverse Transcriptase-Polymerase Chain Reaction and Quantitative Polymerase Chain Reaction of Human Cerebral Organoids

For RT-PCR, the extracted RNAs were reverse transcribed using the protocol supplied with ReverTra Ace qPCR RT Master Mix (FSQ-201; TOYOBO, Osaka, Osaka, Japan). StepOne Plus Real-time PCR System (Thermo Fisher Scientific, Waltham, MA, USA) was used to amplify and quantify levels of target gene cDNA. Quantitative PCR (q-PCR) was conducted using SsoAdvanced Universal SYBR Green Supermix (172-5271; BioRad Laboratories, Hercules, CA, United States) and specific primers for q-PCR. Primers used in this study are listed in Supplementary Table 1. Reactions were run in triplicate. The expression of each gene was normalized to the geometric mean of β-actin (10025636; BioRad Laboratories, Hercules, CA, United States) as a housekeeping gene and analyzed using the ΔΔCT method. Statistical analysis was performed using the GraphPad Prism7 (GraphPad Software). A two-tailed unpaired Student's *t*-test was applied to the q-PCR data. *P*-value < 0.05 was defined as the threshold.

### Tissue-Specific Gene Expression Analysis

Gene expression data for normal human tissues were analyzed from the genotype tissue expression project (GTEx) portal (http://www.gtexportal.org/home/) to compare the similarity of expression pattern of some genes.

### Oxygen-Glucose Deprivation/Reoxygenation of Human Hepatocellular Carcinoma and Quantitative Polymerase Chain Reaction

The HepG2 which were human hepatocellular carcinoma cell line (Cellular Engineering Technologies, Inc., Coralville, IA, USA) were maintained in growth medium (RPMI1640; NACALAI TESQUE, Kyoto, Kyoto, Japan) supplemented with 10% fetal bovine serum (HyClone, SH30396.03, GE Healthcare Life Sciences, Logan, UT, USA), 1% penicillin-streptomycin (26253-84; NACALAI TESQUE, Kyoto, Kyoto, Japan). The cells were incubated at 37°C in 5% CO_2_. Subsequently, cultured HepG2 cells were plated in DMEM without glucose (09891-25; NACALAI TESQUE, Kyoto, Kyoto, Japan), and 1% penicillin-streptomycin (26253-84; NACALAI TESQUE, Kyoto, Kyoto, Japan). Subsequently, the organoids were cultured in a hypoxic incubator (94% N_2_, 5% CO_2_, 1% O_2_) at 37°C for 1 h. For reoxygenation, the medium was changed to growth medium, then incubated (95% air, 5% CO_2_) at 37°C for 1 h. After cell culture, RNA extraction, RT-PCR and q-PCR were performed using the same procedure of human cerebral organoids. The sequences of primers used in this study are listed in Supplementary Table 1.

### Oxygen-Glucose Deprivation/Reoxygenation of Fibroblast, Glioblastoma and Murine Microglia, and Quantitative Polymerase Chain Reaction

Human telomerase reverse transcriptase immortalized fibroblasts (hTERT-BJ), human glioblastoma cell line (U251MG; obtained from Japanese collection of research bioresources) and murine microglial cell line (BV2 cells) were cultured in DMEM with 4.5 g/l glucose (08459-64; NACALAI TESQUE, Kyoto, Kyoto, Japan) supplemented with 10% fetal bovine serum (HyClone, SH30396.03, GE Healthcare Life Sciences, Logan, UT, USA), 1% penicillin-streptomycin (26253-84; NACALAI TESQUE, Kyoto, Kyoto, Japan). The cells were incubated at 37°C in 5% CO_2_. Subsequently, cultured hTERT-BJ, U251MG, and BV2 cells were maintained or plated in DMEM without glucose (09891-25; NACALAI TESQUE, Kyoto, Kyoto, Japan) supplemented with 10% fetal bovine serum (HyClone, SH30396.03, GE Healthcare Life Sciences, Logan, UT, USA), 1% penicillin-streptomycin (26253-84; NACALAI TESQUE, Kyoto, Kyoto, Japan). Hypoxic experiments were performed in a CBS-90 hypoxia workstation (ASTEC, Kasuya, Fukuoka, Japan) at 37°C under severe hypoxic (1% oxygen) or mild conditions (10% oxygen). hTERT-BJ, U251MG, and BV2 cells plated in DMEM with 4.5 g/l glucose (08459-64; NACALAI TESQUE, Kyoto, Kyoto, Japan) or DMEM without glucose (09891-2; NACALAI TESQUE, Kyoto, Kyoto, Japan) were cultured in a hypoxic incubator (94% N_2_, 5% CO_2_, 1% O_2_) at 37°C for 1 h. The mild hypoxic condition we used was 10% oxygen for 24 h. For reoxygenation, the medium was changed to DMEM with 4.5 g/l glucose (08459-64; NACALAI TESQUE, Kyoto, Kyoto, Japan) supplemented with 10% fetal bovine serum (HyClone, SH30396.03, GE Healthcare Life Sciences, Logan, UT, USA), 1% penicillin-streptomycin (26253-84; NACALAI TESQUE, Kyoto, Kyoto, Japan), then incubated (95% air, 5% CO_2_) at 37°C for 1 h. After cell culture, RNA extraction was performed using PureLink RNA Mini Kit (12183020; Thermo Fisher Scientific, Waltham, MA, USA). For RT-PCR, the extracted RNAs were reverse transcribed using the protocol supplied with ReverTra Ace qPCR RT Master Mix (FSQ-201; TOYOBO, Osaka, Osaka, Japan). StepOne Plus Real-time PCR System (Thermo Fisher Scientific, Waltham, MA, USA) was used to amplify and quantify levels of target gene cDNA. Quantitative PCR (q-PCR) was conducted using SsoAdvanced Universal SYBR Green Supermix (172-5271; BioRad Laboratories, Hercules, CA, United States) and specific primers for q-PCR. The sequences of primers used in this study are listed in Supplementary Table 1. Reactions were run in triplicate. The expression of each gene was normalized to thegeometric mean of β-actin as a housekeeping gene and analyzed using the ΔΔCT method. Statistical analysis was performed using the GraphPad Prism7 (GraphPad Software). A two-tailed unpaired Student's *t*-test was applied to the q-PCR data. *P*-value < 0.05 was defined as the threshold.

### Primary Cortical Neuron Culture of Mice

Cortical neurons were prepared from C57BL/6 mice on E16–17. Briefly, the mouse cerebral cortex was digested with 0.25 % trypsin and DNase for 15 min. Cells were passed through a 100 m nylon mesh. The resultant cell suspension was diluted with DMEM/F12 (Thermo Fisher Scientific, Waltham, MA, USA) supplemented with 10% fetal bovine serum (HyClone, SH30396.03, GE Healthcare Life Sciences, Logan, UT, USA) and 1% penicillin-streptomycin (26253-84; NACALAI TESQUE, Kyoto, Kyoto, Japan). Subsequently, the cells were plated on poly-D-lysine coated dishes and maintained at 37°C in 5% CO_2_. The culture medium was replaced with a serum-free DMEM/F12 supplemented with B27 (Thermo Fisher Scientific, Waltham, MA, USA) 24 h after plating.

### Immunocytochemistry of Primary Murine Cortical Neuron Culture

Cultured cortical neurons were fixed with 4% PFA for 1 h at room temperature and incubated with blocking solution containing 1% BSA and 0.1% Triton X-100 in PBS for 1 h. Primary antibodies, anti-TUJ1 (1:1000, 801201; BioLegend, San Diego, CA, USA) was applied overnight at 4°C. Fluorescent dye Alexa Fluor 488-conjugated anti-mouse IgG (715-545-151; Jackson Immunoresearch, West Grove, PA, USA) was used as the secondary antibody.

### Oxygen-Glucose Deprivation/Reoxygenation of Primary Cortical Neuron and Quantitative Polymerase Chain Reaction

The cultured cortical neurons were plated in DMEM without glucose (09891-25; NACALAI TESQUE, Kyoto, Kyoto, Japan), and 1% penicillin-streptomycin (26253-84; NACALAI TESQUE, Kyoto, Kyoto, Japan). Subsequently, the organoids were cultured in a hypoxic incubator (94% N_2_, 5% CO_2_, 1% O_2_) at 37°C for 1 h. For reoxygenation, the medium was changed to Primary Neuron Basal Medium, then incubated (95% air, 5% CO_2_) at 37°C for 1 h. After cell culture, RNA extraction was performed using PureLink RNA Mini Kit (12183020; Thermo Fisher Scientific, Waltham, MA, USA). For RT-PCR, the extracted RNAs were reverse transcribed using the protocol supplied with ReverTra Ace qPCR RT Master Mix (FSQ-201; TOYOBO, Osaka, Osaka, Japan). StepOne Plus Real-time PCR System (Thermo Fisher Scientific, Waltham, MA, USA) was used to amplify and quantify levels of target gene cDNA. Quantitative PCR (q-PCR) was conducted using SsoAdvanced Universal SYBR Green Supermix (172-5271; BioRad Laboratories, Hercules, CA, United States) and specific primers for q-PCR. Primers used in this study are listed in Supplementary Table 1.

Cell culture and hypoxic condition were performed three times, and reactions were run in triplicate. The expression of each gene was normalized to the geometric mean of β-actin as a housekeeping gene and analyzed using the ΔΔCT method. Averaged relative quantification (RQ) was calculated from the average of three RQ values per three samples, every q-PCR. Therefore, one q-PCR demonstrated two averaged RQ values (Ctrl vs. OGD/R), and finally the data from three q-PCR demonstrated six averaged RQ values: three averaged RQ values of controlled samples vs. three averaged RQ after OGD/R. Statistical analysis was performed using the GraphPad Prism7 (GraphPad Software). A two-tailed unpaired Student's *t*-test was applied to the q-PCR data. *P*-value < 0.05 was defined as the threshold.

## Results

### Transcriptome Analysis of Human Brain Ischemic Stroke Model in a Dish

**Figure 1:**
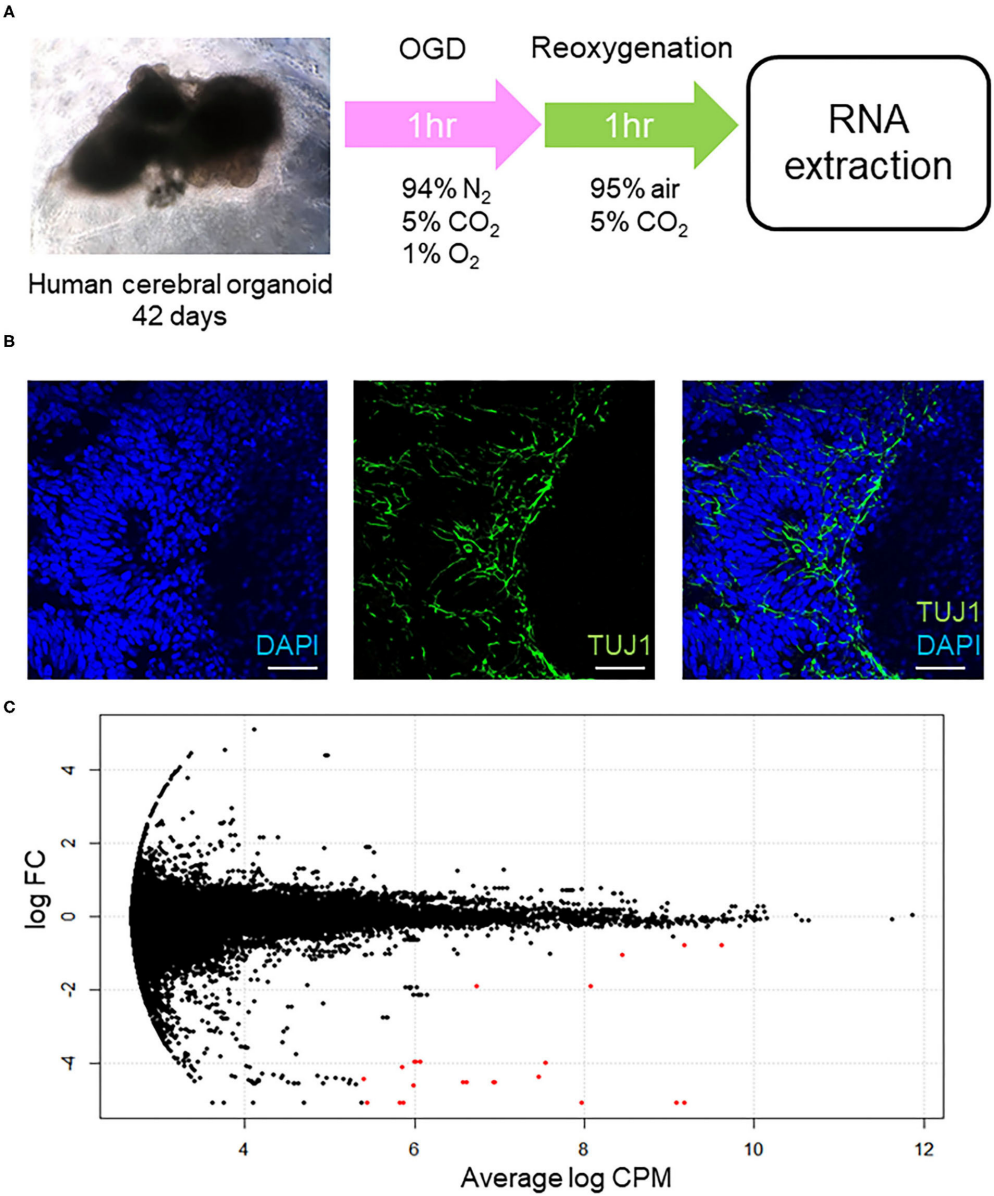
**(A)** Schema of this experimental procedure. First, human cerebral organoids were generated and cultured for 42 days. Subsequently, the organoids were cultured in a hypoxic incubator (94% N_2_, 5% CO_2_, 1% O_2_) at 37°C for 1 h. For reoxygenation, they were incubated (95% air, 5% CO_2_) at 37°C for 1 h. After OGD/R treatment, RNA was extracted. **(B)** Immunohistochemical staining of non-treated human cerebral organoids. Immunohistochemical staining of neuronal cells' marker (TUJ1), and 49,6-diamidino-2phenylindole (DAPI). Fluorescent micrographs show coexisting of TUJ1 (green) and DAPI (blue) at human cerebral organoids. Bars = 100 μm. **(C)** MA plot of cerebral organoids. The X-axis is log CPM, which is a measure of gene expression level. The Y-axis indicates log FC, which is the log difference between cerebral organoids after OGD/R and controls. Red plots are significant DEGs. CPM, counts per million, FC, fold-change; DEGs, differentially expressed genes.

We used six cerebral organoids from one batch and treated three organoids with OGD/R, which is a well-established method of mimicking the pathological processes of ischemia (Hossmann, 1998), to generate a model of cerebral ischemia/reperfusion *in vitro*, and three for normoxic condition. We first cultured the human cerebral organoids for 42 days. Subsequently, these organoids were placed under OGD condition for 1 h and reoxygenation for 1 h. After OGD/R treatment or normoxic condition, RNAs were extracted from each human cerebral organoid prior to RNA isolation and RNA sequencing, of which samples were from every organoid sample, whereupon gene ontology analysis was performed (Figure 1A). Immunohistochemical staining of neuronal cells' marker (TUJ1) and DAPI showed the existence of neuronal cells in our organoids model (Figure 1B). Significant DEGs are colored in red (Figure 1C). Using the threshold of *p* < 0.05 and |log FC| > 0.1, a total of 52 DEGs were determined between intact cerebral organoids and cerebral organoids under OGD/R, including 14 upregulated and 38 downregulated genes (Table 1). When false discovery rate (FDR) adjusted *p*-value ≤ 0.05 was used as a threshold, 15 genes of *AFP, TTR, APOA2, ALB, APOA1, RNA28SN4, APOC3, FTL, AHSG, FGG, FABP1, MIR3615, FGB, RNA45SN4*, and *FGA* were detected, and all genes were found to be downregulated (Table 1). The top five significant downregulated genes were *AFP, TTR, APOA2, ALB*, and *APOA1*, and upregulated genes were *RN7SL2, YWHAE, PTN, NNAT, RN7SL1, POSTN, DNAJB1, MIR6132, LOC105379506*, and *PEA15* (Table 1). In the brain, YWHAE is involved in directing the movement of nerve cells by binding to other proteins (Toyo-oka et al., 2003). Pleiotrophin (*PTN*), which is mainly expressed in the cerebral cortex (Kido et al., 2014; Shen et al., 2017), plays important roles in cell growth and survival, cell migration, and the expression of inflammatory cytokines (Shen et al., 2017), and *POSTN* leads to decreased apoptosis during hypoxia (Aukkarasongsup et al., 2013). These results indicate that upregulated genes might be associated with neuroprotection.

**Table 1:** Profiling gene expression comparing organoids under OGD/R condition with non-treated organoids.

**Gene symbol**	**logFC**	***P*-value**	**FDR**	**Stage**
AFP	−5.05202426	6.61E-89	1.19E-83	Down
TTR	−4.829589384	1.19E-37	7.09E-33	Down
APOA2	−4.375589058	1.38E-24	6.18E-20	Down
ALB	−3.992565039	4.23E-24	1.52E-19	Down
APOA1	−4.544446565	1.37E-17	3.52E-13	Down
RNA28SN4	−1.909649871	2.98E-14	5.94E-10	Down
APOC3	−4.616288539	1.08E-09	1.76E-05	Down
FTL	−0.803043639	2.06E-09	3.08E-05	Down
AHSG	−3.975861808	5.40E-09	7.45E-05	Down
FGG	−4.858272075	7.92E-09	0.0001015	Down
FABP1	−4.10691844	1.31E-07	0.00123622	Down
MIR3615	−1.070468786	1.86E-07	0.00167334	Down
FGB	−4.756570983	2.98E-06	0.02353793	Down
RNA45SN4	−1.899507421	3.02E-06	0.02353793	Down
FGA	−4.451593603	5.72E-06	0.04279685	Down
TF	−4.591735052	2.10E-05	0.14485088	Down
RBP4	−4.460754954	4.01E-05	0.2567811	Down
APOE	−2.151241972	4.23E-05	0.26168457	Down
SELENOP	−2.776594398	4.92E-05	0.29458499	Down
RN7SL2	1.271175267	0.0001831	0.88837764	Down
MIR4709	−1.029200022	0.0002927	1	Down
APOB	−4.704983376	0.0004883	1	Down
GPC3	−1.985098838	0.0005083	1	Down
ALDH1A1	−4.546504241	0.0005188	1	Down
YWHAE	4.380630428	0.0005188	1	Up
PTN	0.775148705	0.0005895	1	Up
s-rRNA	−0.5481881	0.0007606	1	Down
PMEL	−4.567564523	0.0009766	1	Down
NNAT	0.610458501	0.0026105	1	Up
RN7SL1	1.232302556	0.0038383	1	Up
GSTA1	−4.403571625	0.0039063	1	Down
APOA4	−4.361854192	0.0039063	1	Down
POSTN	0.705402287	0.0051026	1	Up
SERPINA1	−3.780463098	0.0063477	1	Down
DNAJB1	1.887700371	0.0066108	1	Up
RNA18SN5	−2.370210664	0.0073853	1	Down
MIR6132	1.736743211	0.0146333	1	Up
LOC105379506	5.83933038	0.015625	1	Up
RBP2	−4.698155314	0.015625	1	Down
DCT	−4.574216819	0.015625	1	Down
AMBP	−3.45106874	0.0214844	1	Down
VTN	−3.157212671	0.0214844	1	Down
H19	−3.053228094	0.0214844	1	Down
PEA15	0.753258601	0.0238198	1	Up
TUBA1A	0.250293775	0.025046	1	Up
MIR1244-1	−0.274612882	0.0268679	1	Down
SPINK1	−4.120454121	0.03125	1	Down
RPS6	−0.293592327	0.0351882	1	Down
MIR4442	0.351338228	0.0366267	1	Up
HSPA8	0.404708278	0.0390529	1	Up
MIR9-2	0.832371451	0.039617	1	Up
TMSB10	0.247345492	0.0457451	1	Up

### Co-expression Network Construction

**Figure 2:**
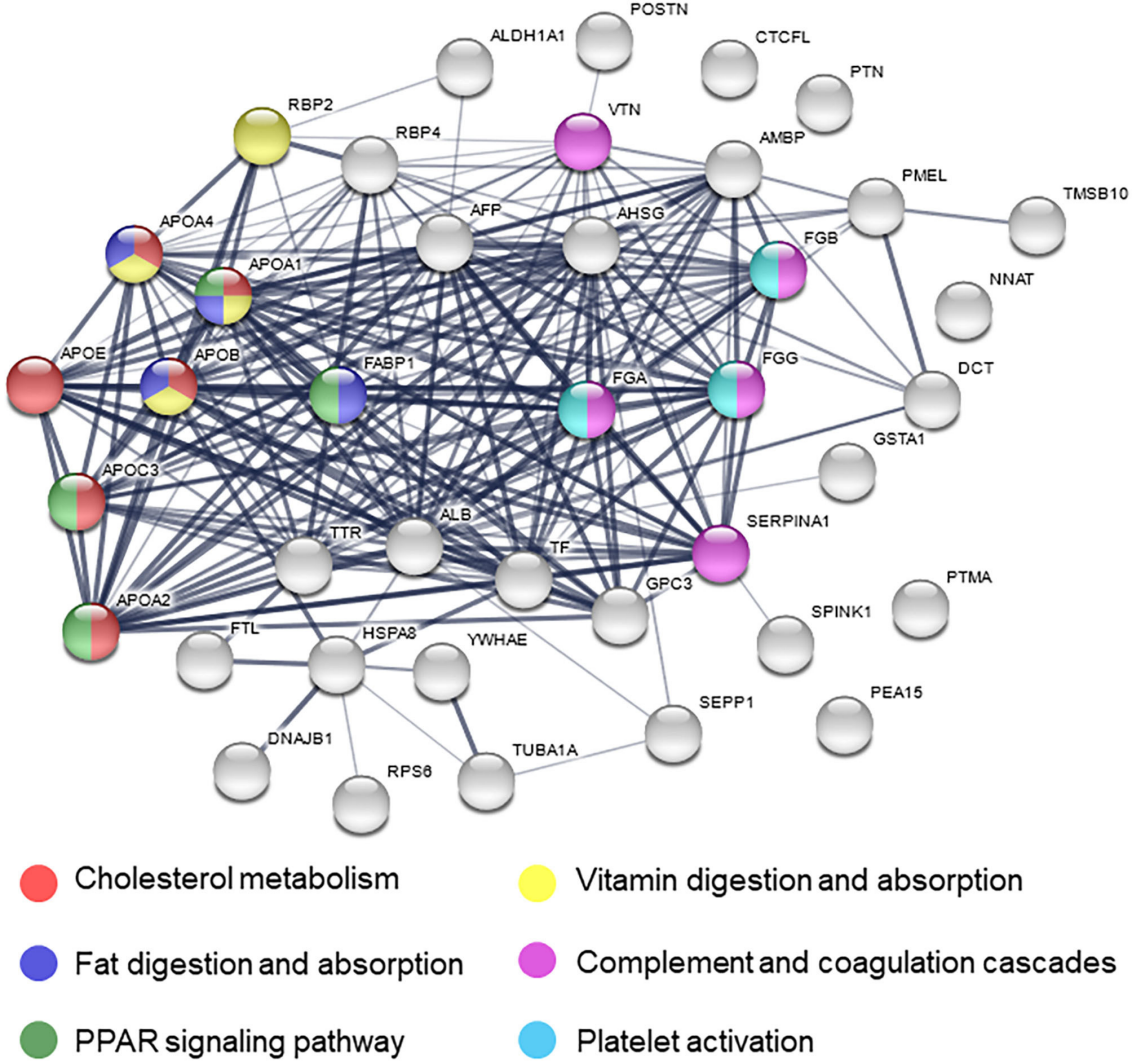
Protein–protein interaction network of differentially expressed genes. Nodes are proteins and the thickness of lines indicates strong interaction between proteins. Colored nodes mean related pathway.

To further investigate pathways involved in ischemic response of human brain, several network analyses were applied. Gene ontological (GO) analysis in the biological process category showed that OGD/R on human cerebral organoids induced lipid metabolism and blood coagulation (Table 2). The pathway-based analysis, KEGG pathway, further highlighted the vitamin digestion and absorption, fat digestion and absorption, peroxisome proliferator-activated receptor (PPAR) signaling pathway, and complement and coagulation cascades (Table 3). Using the STRING online database, 52 DEGs were filtered by the PPI network of DEGs including nodes and edges with parameters of minimum required interaction score >0.4 (medium confidence) (Figure 2). These analyses revealed potential pathways involved in cellular response to ischemic stroke.

**Table 2:** Go term analysis derived from biological process of human cerebral organoids after OGD/R (FDR < 0.05).

**Term**	**Count**	***P*-value**	**FDR**
Retinoid metabolic process	11	5.91E-17	1.55E-13
Platelet degranulation	9	5.07E-11	7.27E-08
Lipoprotein metabolic process	7	1.96E-10	2.81E-07
Cholesterol efflux	6	2.02E-09	2.89E-06
Lipoprotein biosynthetic process	5	2.49E-09	3.58E-06
Phospholipid efflux	5	1.97E-08	2.82E-05
High-density lipoprotein particle remodeling	5	2.68E-08	3.84E-05
Reverse cholesterol transport	5	5.97E-08	8.56E-05
Triglyceride catabolic process	5	2.44E-07	3.50E-04
Cholesterol homeostasis	6	2.72E-07	3.90E-04
High-density lipoprotein particle assembly	4	5.47E-07	7.85E-04
Positive regulation of cholesterol esterification	4	8.20E-07	0.001176
Protein polymerization	4	2.77E-06	0.003979
Negative regulation of very-low-density lipoprotein particle remodeling	3	1.42E-05	0.020297
Cholesterol metabolic process	5	1.47E-05	0.021062
Lipid transport	5	2.28E-05	0.032765
Triglyceride homeostasis	4	2.47E-05	0.035457
Blood coagulation, fibrin clot formation	3	2.83E-05	0.040534

**Table 3:** KEGG pathway of differentially expressed genes.

**Term**	**Count**	**Genes**	**FDR**	***P*-value**
Vitamin digestion and absorption	4	APOA4, APOB, APOA1, RBP2	0.058366266	6.24E-05
Fat digestion and absorption	4	APOA4, APOB, APOA1, FABP1	0.332117918	3.56E-04
PPAR signaling pathway	4	APOA2, APOA1, APOC3, FABP1	1.618503332	0.0017426
Complement and coagulation cascades	4	FGG, FGA, FGB, SERPINA1	1.760575716	0.0018968
Platelet activation	3	FGG, FGA, FGB	54.24273463	0.0801693

### *AHSG, TTR, and FGG* Are Downregulated After OGD/R in the Human Cerebral Organoids

**Figure 3:**
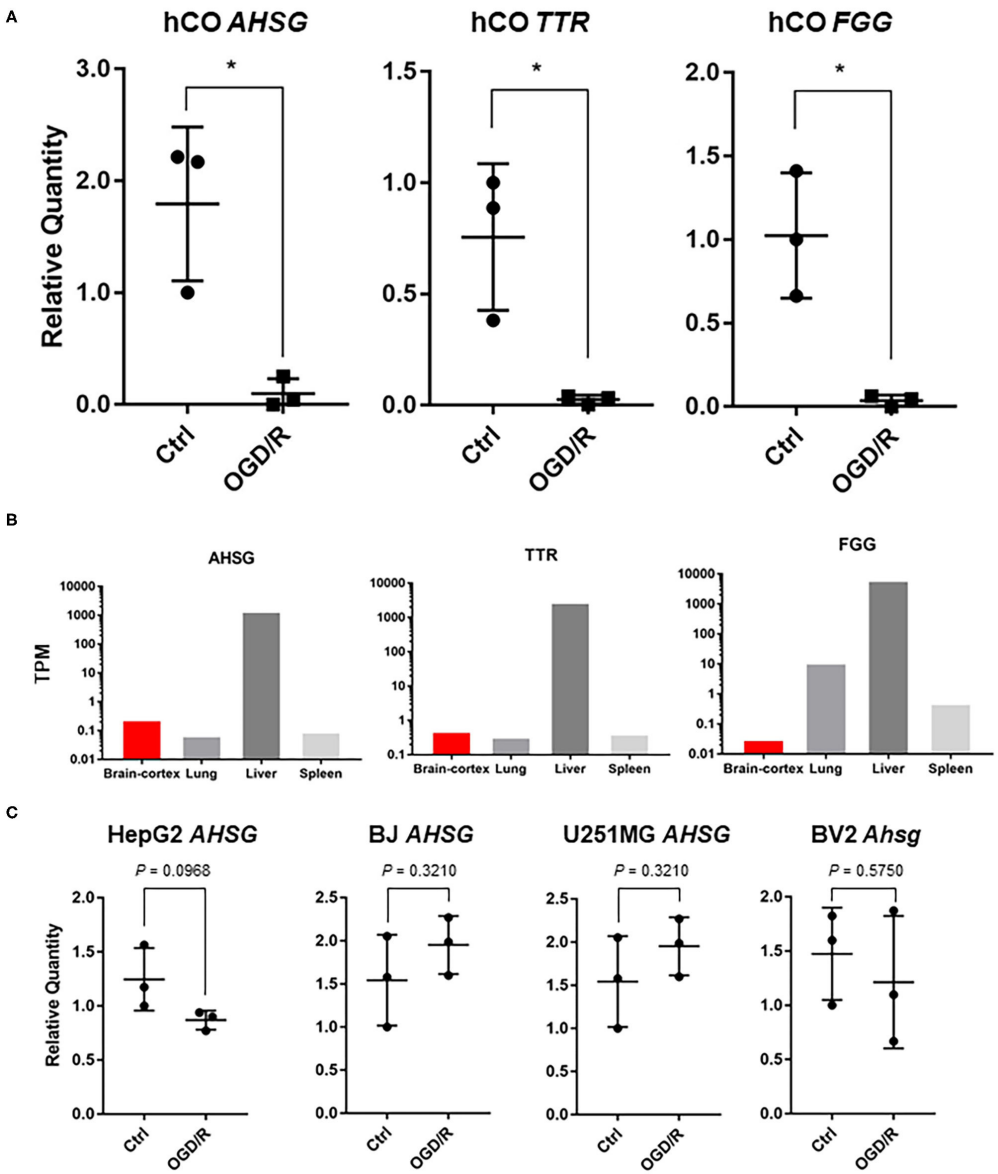
**(A)** qPCR analysis of cerebral organoids [control vs. OGD/R condition]. Gene expression analysis of *AHSG, TTR*, and *FGG* on non-treated organoids (Ctrl) vs. organoids after OGD/R (OGD/R). The *P*-values were 0.0138 (*AHSG*), 0.0185 (*TTR*), and 0.0104 (*FGG*). *P < 0.05 vs. ctrl. **(B)** Tissue-specific gene expression levels using GTEx database. *AHSG, TTR*, and *FGG*. The Y-axis indicates transcripts per million (TPM). **(C)** After OGD/R, expression level of *AHSG*, validated using hepatocellular carcinoma (HepG2), telomerase reverse transcriptase immortalized fibroblasts (hTERT-BJ), glioblastoma (U251MG), and murine microglia (BV2). *TTR* and *FGG* were not detected in BV2 cells and *TTR* was not detected in hTERT-BJ.

Next, we searched for important genes involved in neurological diseases. Among the affected genes after OGD/R in human cerebral organoids, transthyretin (*TTR*), alpha-2-HS-glycoprotein (*AHSG*), and fibrinogen gamma chain (*FGG*) were found to be related to neurological disease. *AHSG* is associated with alopecia-mental retardation syndrome 1 (Reza Sailani et al., 2017), patients of familial amyloid polyneuropathy (*FAP*) carrying *TTR* Met30 mutation (Tanaka et al., 1994), and fibrinogen gamma chain (*FGG*) appear to be protected after ischemic stroke (Cheung et al., 2008). By quantitative PCR (q-PCR), we confirmed downregulation of these three genes; *TTR, AHSG*, and *FGG* were significantly downregulated (Figure 3A). Our results suggest that these three genes have potential to contribute to pathogenesis of ischemic stroke in the human brain.

To find the genes that play more important roles in the brain, we then verified these three genes with gene expression data of normal human tissues obtained from GTEx database. Though a vast majority of AHSG is secreted by the liver in adults (Figure 3B), AHSG is synthesized by various tissues at fetal stage (Stefan et al., 2006; Dabrowska et al., 2015). We demonstrated *AHSG* was downregulated after OGD/R in the human cerebral organoids (Figure 3A). Therefore, to validate *AHSG* expression after OGD/R in non-neural cells, human hepatocellular carcinoma (HepG2), human fibroblasts (hTERT-BJ), and human glioblastoma (U251MG), and murine microglia (BV2 cells) as neural cells were cultured and treated under OGD/R. *Ahsg* or *AHSG* expression of murine microglia, fibroblasts, and glioblastoma was unaltered after OGD/R (Figure 3C). *TTR* and *FGG* were not detected in BV2 cells and *TTR* was not detected in hTERT-BJ (Figure 3C). These results suggest that downregulated *AHSG* of human cerebral organoids might be specific to human fetal brain or human cerebral organoids. *AHSG* expression of hepatocellular carcinoma showed downward trend (1.43 fold; *P* = 0.09) after OGD/R (Figure 3C). This finding might indicate the existence of a cell type with similar gene expression to that of hepatic cells in human cerebral organoids.

### Expression of *FTL* in Human Cerebral Organoids, Murine Cells, and Non-neural Cells

**Figure 4:**
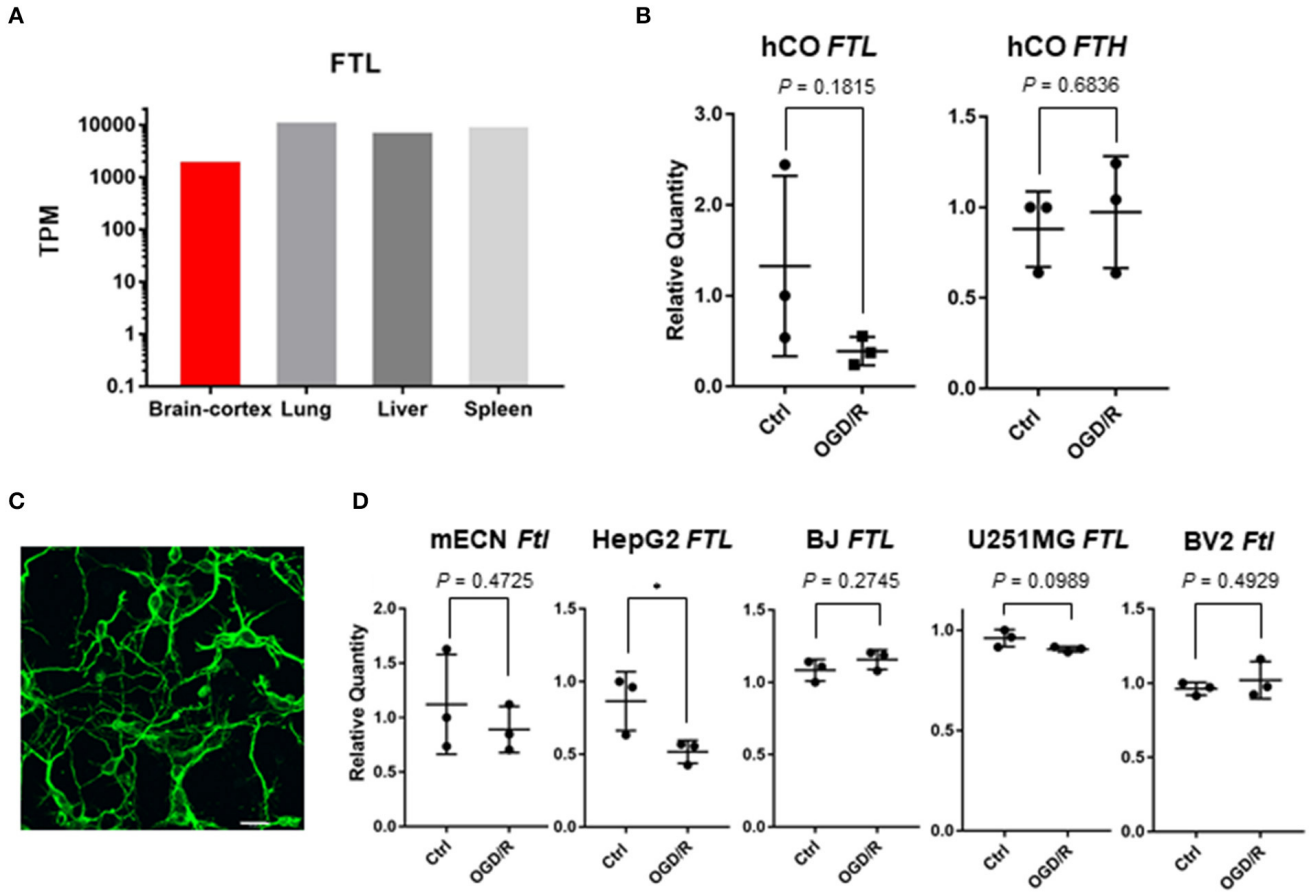
**(A)** Tissue-specific gene expression levels of *FTL* using GTEx database. **(B)** *FTL* and *FTH* expression of human cerebral organoids (hCO) after OGD/R. **(C)** Immunohistochemical staining of mouse embryonic cortical neuron (mECN). Cerebral cortical neurons of mice were immunolabeled for TUJ1 (green). Scale bar = 20 μm. **(D**) *Ftl* or *FTL* expression of mECN, HepG2, hTERT-BJ, U251MG, and BV2 after OGD/R. The *P*-value of HepG2 was 0.0495. *P < 0.05 vs. ctrl.

Ferritin light chain (*FTL*) is gene responsible for neurodegeneration with brain iron accumulation (Muhoberac and Vidal, 2019), and *FTL* was found to be abundantly expressed in the brain (Figure 4A). Although *FTL* was significantly downregulated after OGD/R by using RNA sequencing (Table 1), q-PCR analysis indicated a down-regulated trend of *FTL* (3.40 fold; *P* = 0.18) and no change to the ferritin heavy chain (*FTH*) in the cerebral organoids after OGD/R (Figure 4B). Immunohistochemical staining of neuronal cells' marker (TUJ1) showed the existence of neuronal cells in primary cultured cerebral cortical neurons of mice (Figure 4C). To examine the expression level of *FTL* in mice, the expression level of *Ftl* was tested and unaltered, using primary cultured murine cortical neurons and murine microglia (BV2 cells) treated with OGD/R (Figure 4D). *FTL* expression of OGD/R in non-neural cells, hepatocellular carcinoma (HepG2), telomerase reverse transcriptase immortalized fibroblasts (hTERT-BJ), and glioblastoma (U251MG), were 2D cultured and challenged by OGD/R. While the expression level of *FTL* was unaltered in hTERT-BJ and U251MG, it was downregulated by OGD/R treatment in HepG2 (Figure 4D). Additionally, to analyze the influence of different hypoxic conditions in non-neural cells (hTERT-BJ and U251MG), hTERT-BJ and U251MG cells were 2D cultured and treated by mild hypoxia (i.e., approximately 10% O_2_ for 24 h). The expression level of *FTL* was upregulated by mild hypoxia treatment in both cell lines (Supplementary Figure 1B). In murine primary cultured neurons, fibroblast, and glioblastoma cells, *FTL* expression was not affected by OGD/R as severe hypoxia and might be a reaction specific to human cerebral organoids.

### *PKM2* Is Upregulated by OGD/R in Human Cerebral Organoids and Mouse Cortical Neurons

**Figure 5:**
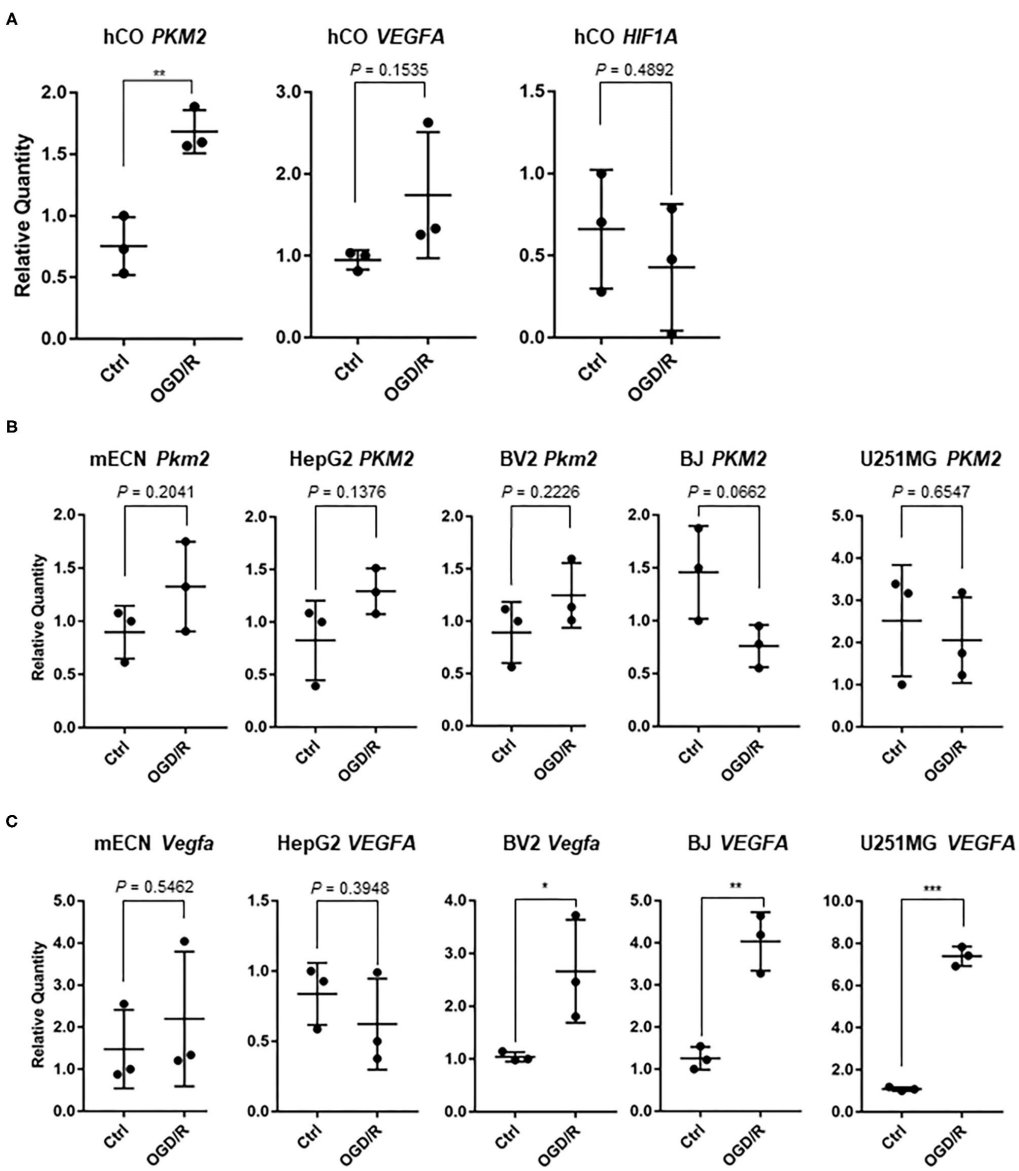
**(A)** In human cerebral organoids (hCO), expression of hypoxic marker genes; *PKM2, VEGFA*, and *HIF1A* after OGD/R. The *P*-value of *PKM2* was 0.0054. **P < 0.001 vs. ctrl. **(B)** After OGD/R, expression level of *PKM2* and *Pkm2* were validated using human cerebral organoids, mouse embryonic cortical neuron (mECN), hepatocellular carcinoma (HepG2), telomerase reverse transcriptase immortalized fibroblasts (hTERT-BJ), and glioblastoma (U251MG). **(C)** After OGD/R, expression level of *VEGFA* and *Vegfa* were validated using human cerebral organoid, mECN, HepG2, hTERT-BJ, and U251MG. The *P*-values were 0.0457 (BV2), 0.0030 (BJ), and < 0.0001 (U251MG). *P < 0.05, **P < 0.001, ***P < 0.0001 vs. ctrl.

**Figure 6:**
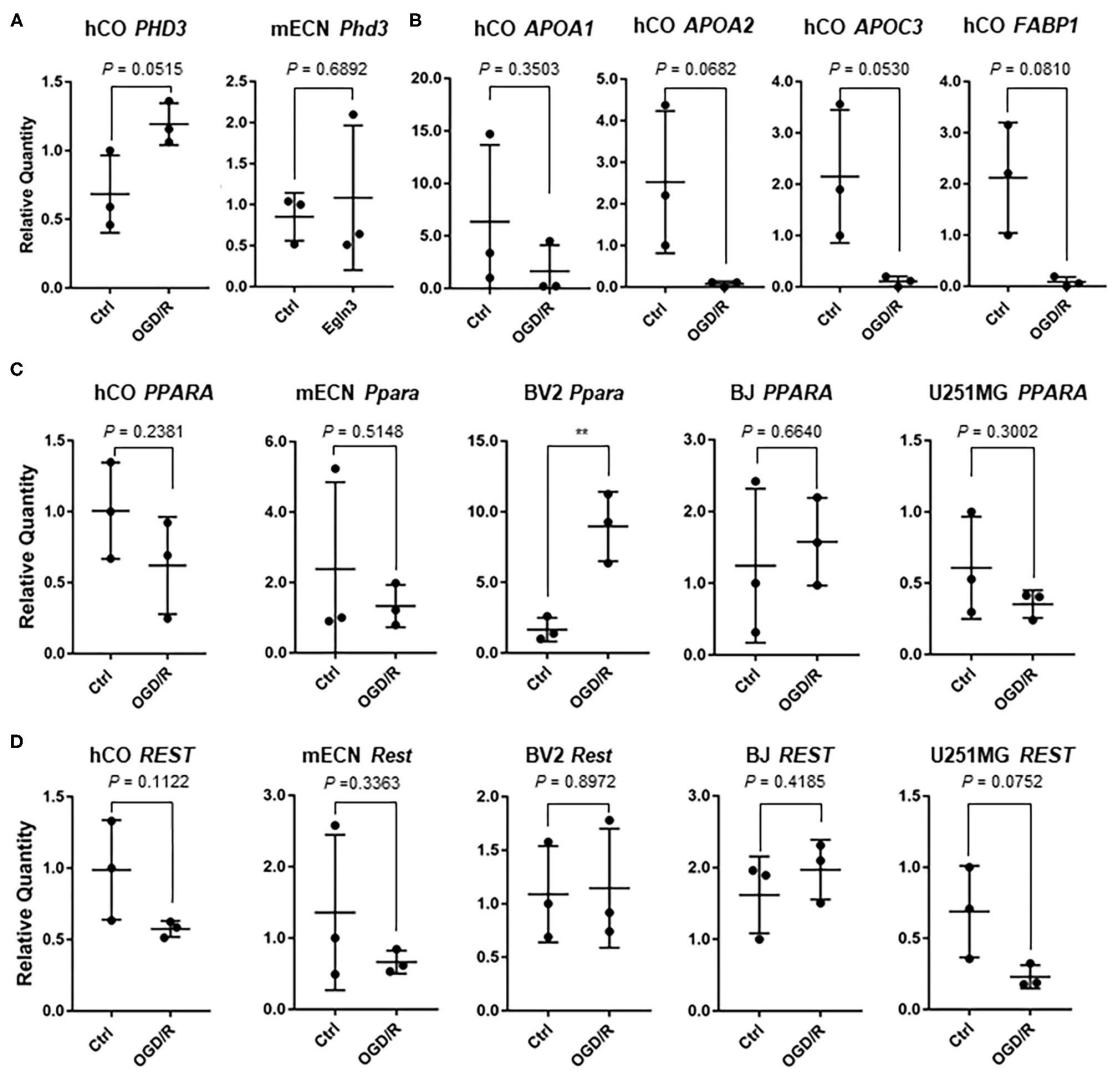
**(A)** Expression of *PHD3* in human cerebral organoid (hCO) and *Egln3* in mECN after OGD/R. **(B)** After OGD/R, expression level of *APOA1, APOA2, APOC3*, and *FABP1* were validated using hCO. **(C)** After OGD/R, expression level of *PPARA* and *Ppara* were validated using hCO, mECN, HepG2, hTERT-BJ, and U251MG. The *P*-value of BV2 was 0.0082. **P < 0.001 vs. ctrl. **(D)** After OGD/R, expression level of *REST* and *Rest* were validated using hCO, mECN, HepG2, hTERT-BJ, and U251MG.

We validated some genes which could be indicators of hypoxia and ischemic markers for cerebral organoids and other cell types. Pyruvate kinase M2 (PKM2), which is associated with glycolytic pathway, converting glucose to lactate and generating ATP (Yang and Lu, 2015), is upregulated after OGD in murine dendritic cells (Jiang et al., 2018). Thus, we searched for important gene associated with hypoxia, and *PKM2* were significantly upregulated after OGD/R in the human cerebral organoids (Figure 5A). Vascular endothelial growth factor (*VEGF*) was relatively upregulated (1.83 fold; *P* = 0.15) and hypoxia inducible factor 1A (*HIF1A*) were not clearly changed after OGD/R in human cerebral organoids (Figure 5A). BV2 cells, hTERT-BJ, U251MG, and HepG2 were cultured and treated under OGD/R. *Pkm2* and *PKM2* did not show significant change after OGD/R (Figure 5B). These results suggest that upregulation of *PKM2* of human cerebral organoids might be specific to humans. *VEGFA* upregulation was significantly verified in hTERT-BJ and U251MG, *Vegfa* was upregulated in BV2 cells, and *Vegfa* and *VEGFA* did not change in murine primary cultured neurons and HepG2 (Figure 5C). We found that the change of hypoxic marker genes independently showed various patterns in human cerebral organoids. We focused on some genes associated with transcriptional factors. Because prolyl hydroxylase 3 (PHD3, encoded by *EGLN3* gene) works as a co-activator of PKM2 and makes the connectivity of HIF1A and PKM2 strong (Luo et al., 2011), we validated expression of *PHD3/Egln3*. *PHD3* was relatively upregulated in organoids (1.74 fold; *P* = 0.05) (Figure 6A). According to pathway analysis of PPAR, we validated relatively downregulated apolipoprotein A1 *(APOA1*) (3.86 fold; *P* = 0.35), apolipoprotein A2 (*APOA2*) (33.28 fold; *P* = 0.06), apolipoprotein C3 (*APOC3*) (20.12 fold; *P* = 0.05), and fatty acid binding protein 1 (*FABP1*) (25.20 fold; *P* = 0.08) (Figure 6B). The expression level of *PPARA* as a nuclear transcription factor was upregulated in BV2 cells and was not altered in other cell types (Figure 6C). Because the RE1-silencing transcription factor (*REST*) as another nuclear transcription factor works against the oxidative stress of aging brain and Alzheimer's Disease using immunostaining of human brain (Lu et al., 2014), we tested the expression level of *REST* and it was not clearly changed (Figure 6D).

## Discussion

*In vivo* models reflect many factors including central nervous system, vascular system, and immune systems, and the interaction and physiological systems make interpretations of the results complex. On the contrary, *in vitro* 2D models consist of homotypic cell populations without cellular diversity and are unlikely to represent tissue architecture of actual tissues. Therefore, development and application of *in vitro* 3D model organoids are required to interpret the actual organ systems and cellular reactions. Additionally, *in vitro* maturation of human iPSC-derived 3D organoids could be a useful model for studying the pathophysiology of disease. Some genes of 3D cultured chondrocytes are highly increased and other genes are decreased compared with 2D models (Caron et al., 2012), and kidney organoids are more sensitive to nephrotoxicity than 2D cultured cell lines (Astashkina et al., 2012). Although human cerebral organoids lack fully matured neuronal tissue, our model still provides a biologically plausible means to study ischemic injury response in the human brain tissue. By using human cerebral organoids combined with series of transcriptome analysis, we revealed that *TTR, AHSG*, and *FGG* were downregulated after OGD/R. Although we were able to detect a downregulation of *FTL* in human cerebral organoids after OGD/R by using RNA-seq, we found no significant change in q-PCR. Simultaneously we validated unaltered *FTL* in non-neural cells (fibroblasts and glioblastoma), murine microglia, and primary cultured murine cortical neurons after OGD/R. A larger sample size of cerebral organoids is preferable for validating *FTL* expression level because there are differences in maturity among individual organoids. Although *FTL* was more expressed than *AHSG, TTR*, and *FGG* using gene expression data for normal human tissues (Figures 3B, 4A), it remains debatable whether iron itself and iron accumulation were beneficial or harmful after experimental stroke. Some groups have shown that brain damage after ischemia is correlated with overloaded iron (Castellanos et al., 2002; Mehta et al., 2004), while others suggest that iron storage is not evil (Christensen et al., 2002; Millerot et al., 2005). Therefore, further investigations are required to clarify the role of iron and iron storage after ischemic stroke.

We revealed that *FTL, TTR, AHSG*, and *FGG* are downregulated after OGD/R, but the distribution of FTL, TTR, AHSG, and FGG in human tissue is independent (Figures 3B, 4A). While FTL, TTR, AHSG, and FGG were abundantly expressed in liver, TTR, AHSG, and FGG were not sufficient in brain (Figures 3B, 4A). To validate the reaction of hepatocytes, we used HepG2 cells and *FTL* and *AHSG* tended to downregulate after OGD/R (Figures 3C, 4D). It could be possible that hepatocytes or hepatocyte-like cells co-exist in the human cerebral organoids. Although cell types of the human cerebral organoids are derived from ectodermal origin (Quadrato et al., 2017), hepatic cells are derived from endoderm origin. There might be co-existing hepatic cells or hepatocyte-like cells in the human cerebral organoids we used in this study, and this might be potential limitations of the current study.

We revealed the expression level of *PKM2* of the human cerebral organoids after OGD/R was predominantly elevated. On the contrary the expression level of *PKM2* of other cells after OGD/R was not evidently changed. *Pkm2*, both mRNA and protein, is upregulated after OGD in murine dendritic cells, which are immune-modulatory cells that do not exist in our human cerebral organoids (Jiang et al., 2018). These results suggest that the upregulation of *PKM2* might be useful as a hypoxic marker in human cerebral organoids, while an expression of *PKM2* might be controversial in other cell types. Although PKM2 plays protective role in acute phase of focal ischemic stroke model of mice (Chen et al., 2018), loss of Pkm2 using *Pkm2* knockout mice also protects ischemic tissues by maintaining mitochondrial biogenesis (Hauck et al., 2020). Thus, the role of *PKM2* upon OGD/R insults is still controversial. *PKM2* is a target gene of HIF-1α, regulates gene expression of proteins associated with the glycolytic pathway and can accelerate metabolic reprogramming (Luo et al., 2011). Furthermore, *PHD3/Egln3* induced by hypoxia works as a co-activator of *PKM2* by HIF pathway (Pescador et al., 2005). *PHD3/Egln3* is relatively upregulated after OGD/R. This might accelerate metabolic switch and lead to decreased high-density lipoproteins (HDLs) including *APOA1, APOA2*, and *APOC3*, all of which showed a trend of downregulation upon OGD/R (Figure 6B). Therefore, PKM2 may act synergistically with PHD/Hif-1α in our OGD/R model. On the other hand, although some genes about fatty acid were relatively downregulated (Figure 6B), *PPARA* downregulation was validated only in murine microglia (Figure 6C). The reason why *PPARA* was unchanged in cerebral organoids might be due to a lack of microglia derived from mesoderm. Novel system of cerebral organoids including microglia will resolve this limitation. REST is a transcriptional factor as a silencer, concerning neurogenesis, neuronal differentiation, and growth (Paquette et al., 2000). We validated relatively downregulated *REST* in cerebral organoids, not murine primary cultured neurons (Figure 6D). Because some genes about neuronal movement, growth, and survival were upregulated after OGD/R using RNA-seq data set, *REST* downregulation might lead to neurogenesis and neuronal differentiation. These results could be specific for 3D embryonic culture system. PKM2 is inhibited by PPARγ in two breast cancer cell lines, and this inhibition might decrease ATP levels and avoid apoptosis (Shashni et al., 2013). We showed PPAR signaling pathway is associated (Figure 2) and *PKM2* was upregulated after OGD/R in human cerebral organoids (Figure 5A). This could suggest that PPARγ is downregulated and *PKM2* is upregulated subsequently after ischemia. The PPARγ agonist conducts neuroprotection against focal ischemia in the rat brain (Zhao et al., 2005). Dual PPARα/γ agonist improves stroke outcome after transient cerebral ischemia in mice (Boujon et al., 2019). Therefore, therapeutic agents related lipid metabolism, including PPAR agonist, may be effective for ischemia.

Our study did not clarify either the change of common hypoxic marker genes (*VEGF* and *HIF1A*) of human cerebral organoids occurs after OGD/R (Figures 5A,C) or a change in other hypoxic marker genes (*LDHA, CA9*, and *PGK1*) (Supplementary Figure 1C). Lacking vascular system, brain organoids necrotize in their core as they grow and exist around the core healthy tissues *in vitro* (Lancaster, 2018). To resolve the problem of central core necrosis of brain organoids, they are transplanted into the mouse brain as vascularized model of brain organoids *in vivo* (Mansour et al., 2018). *In vitro* organotypic slice culture improved long-term survival (Giandomenico et al., 2021), and air–liquid interface culture for cerebral organoids improved survival, axon outgrowth, and decreased apoptosis rate (Giandomenico et al., 2019). It might be the reason why *VEGFA* and *HIF1A* did not significantly change, and the lack of vascular system was one of the limitation of cerebral organoids. Although *VEGF* and *HIF1A* were not markedly upregulated, we validated upregulated *HSPA8*, stress-induced protein: heat-shock cognate protein 70 (HSC70) coding gene, using RNA-seq (Table 1). Because HSC70 assembles after cerebral ischemia of rats (Hu et al., 1998) and HSC70 involves diverse proteostasis mechanisms including chaperone mediated autophagy which contributes to oxidative stress response (Kiffin et al., 2004; Loeffler et al., 2016), upregulated *HSPA8* coding HSC70 indicated the oxidative stress of our model was existing.

There are a number of groups trying to apply organoid system to study isolated organ model in a dish. For example, James Hudson and colleagues showed that mild-hypoxia and/or metabolic changes are critical environmental triggers for maturation of cardiac cells using human cardiac organoids (Mills et al., 2019). In their experiments, they treated the human cardiac organoids by changing the serum free media, lowering glucose concentration, and increasing the concentration of fatty acid. Palmitate was used as a fatty acid substrate, as it is one of the most abundant fatty acids circulating during the neonatal period (Bougnères et al., 1982). Importantly, Hesham Sadek and colleagues discovered mild-hypoxia to be a stimulus for the metabolic switch from carbohydrates to fatty acids in a neonatal mouse cardiac tissue (Puente et al., 2014) and adult mouse cardiac tissue (Nakada et al., 2017), and therefore human cardiac organoids could model the postnatal switch in metabolic substrates from carbohydrates to fatty acids. In our experiment, we removed glucose and oxygen without substituting fatty acid. Thus, the human brain organoids do not get substrates even if the gene expression may alter metabolic switch from carbohydrates to fatty acids. The difference of *FTL* expression between mild-hypoxia and severe-hypoxia Figure 4D, Supplementary Figure 1B might suggest mild-hypoxia tends to metabolize fatty acid, compared with severe-hypoxia. Taken together, our experimental condition is not comparable to environmental changes that induce postnatal metabolic shift; instead, it mimics ischemic tissue. More detailed time course about hypoxia will be needed for further investigation in the future. It is possible that neural cells in the brain and cardiac muscle cells in the heart may respond differentially to severe hypoxia. Therefore, in order to adapt their findings from the cardiac organoids (Mills et al., 2017) to our brain organoids, neural cells would need to have similar capability to respond to same environmental stimuli. Further investigations are required to understand how neural and non-neural cells in the brain respond to pathogenic severe hypoxia.

Our goal here is to extract the direct effects on neural tissues out of spatio-temporally complex system organized by various cell types. Our cerebral organoids are free from the effect of blood vessels and blood-derived inflammatory cells invading through blood vessels. Therefore, our experimental model enabled us to evaluate the direct effect of deprivation of both oxygen and glucose followed by reoxygenation on brain tissue. Although we are aware of the limitations of applying the human cerebral organoid as a model for human ischemic stroke, there are expectations for the potential development and advancement for further applications of our model.

## Data Availability Statement

The datasets presented in this study can be found in online repositories. The names of the repository/repositories and accession number(s) can be found below: https://www.ddbj.nig.ac.jp/, DRA010225.

## Ethics Statement

The animal study was reviewed and approved by Animal Care Committee of Nara Medical University.

## Author Contributions

NIw, TM, KSu, and EM designed the study. NIw, TM, NIg, KK, NM, YMS, TS, SKo, TT, MN, and RN conducted the research. NIw, TM, NIg, KK, YMS, MM, NE, TK, TI, KSa, HK, YS, WK, AW, YN, EM, and KSu analyzed the data. NIw, TM, TT, KSu, and EM wrote the paper. All authors contributed to manuscript revision and read and approved the submitted version.

## Conflict of Interest

The authors declare that the research was conducted in the absence of any commercial or financial relationships that could be construed as a potential conflict of interest.

## Supplementary Material

Supplementary Figure 1 **(A)** Immunohistochemical staining of mouse embryonic cortical neuron (mECN). Cerebral cortical neurons of mice were immunolabeled for TUJ1 (green). Scale bar = 100 μm. **(B)** Expression level of *Ftl* and *FTL* under mild hypoxia (i.e., O_2_ 10% for 24 h). **(C)** In hCO, expression of other hypoxic marker genes; *LDHA, CA9*, and *PGK1* after OGD/R.

Supplementary Table 1 Primer pairs for qPCRs used in this study.
