# Supplementary figures and images for: Gene Expression Profiles of Human Cerebral Organoids Identify PPAR Pathway and PKM2 as Key Markers for Oxygen-Glucose Deprivation and Reoxygenation
Source: Front Cell Neurosci. 2021 Jun 8;15:605030. doi: 10.3389/fncel.2021.605030 (PMC8217463; doi:10.3389/fncel.2021.605030)

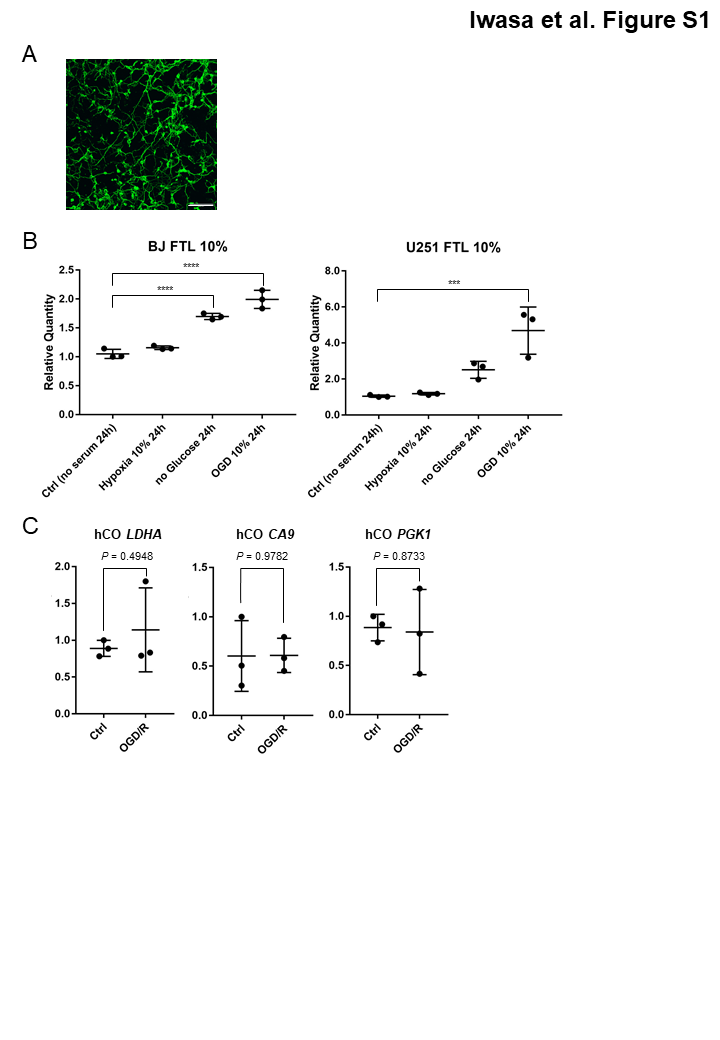

Supplement: Supplementary Figure 1 — (A) Immunohistochemical staining of mouse embryonic cortical neuron (mECN). Cerebral cortical neurons of mice were immunolabeled for TUJ1 (green). Scale bar = 100 μm. (B) Expression level of Ftl and FTL under mild hypoxia (i.e., O2 10% for 24 h). (C) In hCO, expression of other hypoxic marker genes; LDHA, CA9, and PGK1 after OGD/R. [file Image_1.TIF]
